# Increased epithelial membrane protein 2 expression in glioblastoma after treatment with bevacizumab

**DOI:** 10.1093/noajnl/vdaa112

**Published:** 2020-09-08

**Authors:** Kunal S Patel, Sameer Kejriwal, Samasuk Thammachantha, Courtney Duong, Adrian Murillo, Lynn K Gordon, Timothy F Cloughesy, Linda Liau, William Yong, Isaac Yang, Madhuri Wadehra

**Affiliations:** 1 Department of Neurosurgery, University of California Los Angeles, Los Angeles, California, USA; 2 Department of Pathology and Laboratory Medicine, University of California Los Angeles, Los Angeles, California, USA; 3 Jonsson Comprehensive Cancer Center, University of California Los Angeles, Los Angeles, California, USA; 4 Department of Ophthalmology, University of California Los Angeles, Los Angeles, California, USA

**Keywords:** angiogenesis, bevacizumab, EMP2, glioblastoma

## Abstract

**Background:**

Antiangiogenic therapy with bevacizumab has failed to provide substantial gains in overall survival. Epithelial membrane protein 2 (EMP2) is a cell surface protein that has been previously shown to be expressed in glioblastoma, correlate with poor survival, and regulate neoangiogenesis in cell lines. Thus, the relationship between bevacizumab and EMP2 was investigated.

**Methods:**

Tumor samples were obtained from 12 patients with newly diagnosed glioblastoma at 2 time points: (1) during the initial surgery and (2) during a subsequent surgery following disease recurrence post-bevacizumab treatment. Clinical characteristics and survival data from these patients were collected, and tumor samples were stained for EMP2 expression. The IVY Glioblastoma Atlas Project database was used to evaluate EMP2 expression levels in 270 samples by differing histological areas of the tumor.

**Results:**

Patients with high EMP2 staining at initial diagnosis had decreased progression-free and overall survival after bevacizumab (*median progression-free survival 4.6 months vs 5.9 months; log-rank P = .076 and overall survival 7.7 months vs 14.4 months; log-rank P = .011*). There was increased EMP2 staining in samples obtained after bevacizumab treatment in both unpaired (*mean H-score 2.31 vs 1.76; P = .006*) and paired analyses (*mean difference 0.571; P = .019*). This expression increase correlated with length of bevacizumab therapy (*R*^2^  *= 0.449; Pearson P = .024*).

**Conclusions:**

Bevacizumab treatment increased EMP2 protein expression. This increase in EMP2 correlated with reduced mean survival time post-bevacizumab therapy. We hypothesize a role of EMP2 in clinical bevacizumab resistance and as a potential antiangiogenic therapeutic target in glioblastoma.

**Key Points**

- EMP2 expression was associated with survival after bevacizumab therapy.
- EMP2 expression increased after bevacizumab treatment in glioblastoma.

**Importance of the Study**

We have previously established EMP2 as a proangiogenic protein in glioblastoma. In this study, we further characterize the biology of EMP2 in glioblastoma by evaluating protein expression in matched clinical glioblastoma samples prior to and after bevacizumab treatment. In addition, we investigate localization of the EMP2 in glioblastoma and its effect on survival in regard to bevacizumab. Overall, we supply data EMP2 to potentially implicate a prognostic and antiangiogenic therapeutic target for glioblastoma.

A pathologic hallmark and diagnostic criterion of glioblastoma is the recruitment and proliferation of blood vessels to facilitate further tumor growth.^1,2^ Given this association, antiangiogenics have been investigated as a potential therapeutic strategy for glioblastoma. The first and best characterized antiangiogenic strategy has been the use of bevacizumab,^3,4^ an antibody that binds and inactivates vascular endothelial growth factor A (VEGF-A).^5^ In addition, the following alternative strategies for modulating angiogenesis through the VEGF pathway have been investigated: (1) aflibercept (VEGF trap), a decoy receptor for VEGF-A and B,^6^ (2) anti-VEGF receptor antibodies,^7,8^ and (3) intracellular tyrosine kinase inhibitors (cediranib and vandetanib) blocking downstream signaling.^9–12^ There are several randomized controlled trials studying these anti-VEGF agents in glioblastoma.^13–18^ In robust meta-analyses of these data, the overall data suggest a progression-free survival but not overall survival benefit of antiangiogenic therapy as adjuvant treatment in new or recurrent glioblastoma, with or without concurrent chemotherapy.^19^ Given these data, it is clear that targeting angiogenesis in glioblastoma with a monotherapy targeting the VEGF pathway is insufficient, and thus, there is an urgent need to identify novel targets that can predict therapeutic response or be used in concert to improve results.

We have previously identified epithelial membrane protein 2 (EMP2) as a cell surface protein present in a number of tumors including glioblastoma but not in nonpathologic brain tissue.^20,21^ In in vitro and in vivo models of glioblastoma, EMP2 expression was associated with increased tumor size, vascularity, and VEGF-A expression.^22^ Furthermore, humanized monoclonal anti-EMP2 antibody leads to significant decreases in tumor load and angiogenesis.^22^ These experimental data suggest a potential antiangiogenic target in EMP2. In this study, we evaluate the relationship between EMP2 and bevacizumab in a series of clinical glioblastoma specimens, and we identify increased EMP2 expression in tumor samples after bevacizumab treatment, proportional to the length of bevacizumab treatment. These clinical data supplement our existing data suggesting EMP2 as a potential therapeutic target in glioblastoma.

## Methods and Materials

### Patient Selection

This study was approved by the institutional review board and informed research consent was acquired in all cases. Tumor tissue was prospectively collected for patients undergoing surgery for suspected glioblastoma. Inclusion criteria for this study included (1) adult patients, (2) patients with new tumors, (3) pathologically proven WHO IV glioblastoma, (4) acquired tumor tissue both prior to and after bevacizumab treatment, and (5) clinical and radiologic follow-up of at least 3 months. After the implementation of the above inclusion criteria, 12 patients were included in this study.

### The Cancer Genome Atlas and IVY Glioblastoma Atlas Project Data

Using the The Cancer Genome Atlas (TCGA) Data Portal (http://tcga-data.nci.nih.giv/tcga/), robust multichip average normalized mRNA expression data from the Affymetrix HG-U133A Gene Chip was downloaded along with associated survival data and genomic subtype data.^23,24^ The IVY Glioblastoma Atlas Project (IVY GAP) Data Portal (http://glioblastoma.alleninstitute.org/)^25^ was used to collect RSEM normalized RNA sequencing data along with histological annotation of the sample source.

### Immunohistochemistry

Paraffin-embedded clinical tumor samples and control tissue were sliced into 5-µm-thick samples to be used for immunohistochemistry (IHC). First, sections were deparaffinized in 3 washes of xylene and then rehydrated in serial dilutions of ethanol. Antigen retrieval was performed by placing the slides in a container of 0.1 mol/L citrate, pH 6.0, at 95°C for 25 min. Sections were incubated with polyclonal rabbit antisera against human EMP2,^26^ and staining visualized using DAKO HRP Anti-Mouse followed by DAB (3,3′-diaminobenzidine) as the substrate. Counterstaining was performed with hematoxylin. Slides were then dehydrated and mounted. An isotype control (rabbit sera) was used as a negative control.

IHC staining was evaluated by a neuropathologist (S.T.), blinded to patient outcomes, based on both the staining intensity (0 = no detection; 1 = weak; 2 = moderate; 3 = strong) and the positive staining percentage (0–100%). The product of the 2 values was used to generate a histologic score (H-score).

### Statistical Analysis

Statistical analysis was carried out on GraphPad Prism v6.0h (GraphPad Software, Inc.). Kaplan–Meier survival analysis was carried out using the dependent variables progression-free survival, surgery-free survival, and overall survival stratified by high and low EMP2 H-score from both a clinical cohort and TCGA cohort. Linear regression analysis was implemented to evaluate the relationship between the length of bevacizumab treatment and EMP2 H-score. Unpaired and paired *t*-tests with Welch’s correction for unequal variance were used to evaluate differences in EMP2 H-score before and after bevacizumab treatment. Errors are presented as the standard error of the mean (SEM) or standard deviation (SD).

## Results

### EMP2 Is Localized to Areas of Vascular Proliferation in Glioblastoma

**Figure 1.**
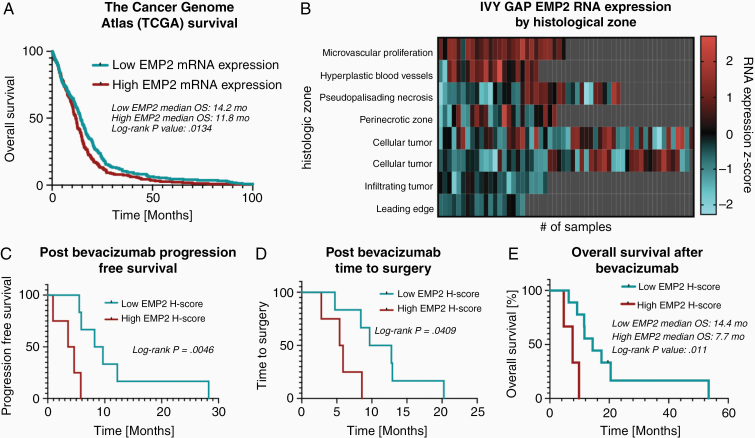
Increased EMP2 is associated with decreased survival after bevacizumab therapy. The Cancer Genome Atlas (TCGA) database was queried for EMP2 RNA expression data and specimens were stratified by high or low EMP2 expression. Kaplan–Meier analysis using the TCGA dataset (A). The IVY Glioblastoma Atlas Project (IVY GAP) database was used to identify RNA expression through normalized z-score at different areas of the tumor as defined by tumor histology (B). Kaplan–Meier analysis using clinical data for progression-free survival (C), time to repeat surgery (D), and overall survival (E), after bevacizumab therapy was initiated.

We have previously published the expression pattern of EMP2 using a tissue microarray containing 110 patients diagnosed with glioblastoma and shown that high levels correlate with poor survival.^20^ To expand on this initial observation, the TCGA dataset was queried for RNA expression data and overall survival for patients with glioblastoma. In 525 patients stratified by median EMP2 mRNA expression, there was decreased overall survival in patients with high EMP2 mRNA expression (*median survival 11.8 months vs 14.2 months; log-rank P = .0134*; Figure 1A).

Our previous studies suggested a role for EMP2 in regulating neoangiogenesis in glioblastoma cell lines.^20,22^ In order to extend this observation to patient tumors, the IVY GAP Database was queried for RNA sequencing data on EMP2 in clinical glioblastoma specimens. Z-score-normalized RNA expression values were obtained from a total of 270 samples from 37 tumors. Each sample had an associated histological identifier, corresponding to the pathological description of the region from which the sample was obtained. These included areas of microvascular proliferation, hyperplastic blood vessels, pseudopalisading necrosis, perinecrotic zone, cellular tumor, infiltrating tumor, and the leading edge of the tumor. A heat map was created from RNA expression values by histological identifier and there was increased EMP2 RNA expression in areas of microvascular proliferation and hyperplastic blood vessels relative to other areas of the tumor (Figure 1B).

### Patient Characteristics

The data above collectively suggested that EMP2 may help regulate neoangiogenesis within the tumor parenchyma as well as tumor cell invasion along the leading edge. Given the widespread usage of antiangiogenics in glioblastoma treatment, we examined the effects of bevacizumab on EMP2 levels. We hypothesized that bevacizumab treatment, a monoclonal antibody against soluble VEGF, may correlate with increased EMP2 levels given the similarities observed with EMP2 upregulation and bevacizumab resistance.^22,27^

A total of 12 patients with tissue samples from surgical resection at our institution between 2003 and 2013 at the following time points (1) upon initial presentation and (2) after bevacizumab treatment were included in this study. Clinical and tumor characteristics are included in Table 1. There were 8 (67%) males and 4 (33%) females. The average age was 55 years (range 46–73 years). Tumor locations included were temporal for 5 (42%), frontal for 4 (33%), and parietal for 3 (25%). Gross total resection was achieved in 10 (83%) patients. Two tumors (17%) had EGFR amplifications and 1 tumor (8%) had an IDH1 R132H mutation. All patients had newly diagnosed tumors initially treated with surgical resection and adjuvant temozolomide and radiation therapy. Upon subsequent recurrence, all patients had bevacizumab with or without additional chemotherapy. Additional therapy included 1 patient with aflibercept (VEGF trap) prior to bevacizumab treatment, 1 patient with ofranergene obadenovec (VB-111) after bevacizumab treatment, and 2 patients with dendritic cell vaccine (DCvax-L) after bevacizumab treatment. Average length of bevacizumab treatment was 9.5 weeks (range 4–24 weeks). Average progression-free survival after bevacizumab treatment was 6.4 months (range 1–13 months). Average time to surgery after bevacizumab treatment was 7.7 months (range 3–13 months). Overall survival after bevacizumab treatment was 15.5 months (range 5–53 months) and overall survival from initial diagnosis was 39 months (range 10–147 months).

**Table 1.** Patient Characteristics

Characteristic	*N* = 12
Age (years)	55 (range 46–73 years)
Gender	
Male	8 (67%)
Female	4 (33%)
Tumor location	
Temporal	5 (42%)
Frontal	4 (33%)
Parietal	3 (25%)
Gross total resection	10 (83%)
EGFR amplifications	2 (17%)
IDH1 R132H mutation	1 (8%)
Additional chemotherapy	
Aflibercept (VEGF trap)	1 (8%)
Ofranergene obadenovec (VB-111)	1 (8%)
Dendritic cell vaccine (DCvax-L)	2 (17%)
Bevacizumab treatment	
Treatment length	9.5 (range 4–24 weeks)
Progression-free survival after treatment	6.4 (range 1–13 months)
Time to surgery after treatment	7.7 (range 3–13 months)
Survival after treatment	15.5 (range 5–53 months)
Survival from the initial diagnosis	39 (range 10–147 months)

### EMP2 Expression Is Associated With Decreased Survival With Bevacizumab Treatment

Twelve patients had tumor samples from newly diagnosed glioblastoma available for IHC analysis. The average staining intensity (scored from 0 to 3) was 2.1 (*SD* = *0.43; range 1–3*), the average percent staining was 85% (*SD = 12.1; range 60–100*), and the average H-score was 183 (*SD = 55; range 112.5–300*). A cutoff of H-score at least 200 was used to stratify high initial EMP2 from low initial EMP2 levels for further analyses. There was no difference in clinical characteristics between these 2 groups including age (*57.6 vs 51.8; P = .29*), gender (*29% female vs 40% female; P = .71*), or gross total resection rate (*86% vs 80%; P = .82*). To investigate the interaction between EMP2 levels and response to bevacizumab treatment, we evaluated survival after bevacizumab treatment stratified by EMP2 H-score. In patients with a high EMP2 H-score, there was decreased progression-free survival after bevacizumab (*median survival 4.6 months vs 5.9 months; log-rank P = .0046*; Figure 1C), decreased time to repeat surgery (*median length 5.9 months vs 8.4 months; log-rank P = .0409*; Figure 1D), and decreased overall survival (*median survival 7.7 months vs 14.4 months; log-rank P = .011*; Figure 1E).

### EMP2 Expression Increases After Bevacizumab Treatment in Clinical Glioblastoma Specimens

**Figure 2.**
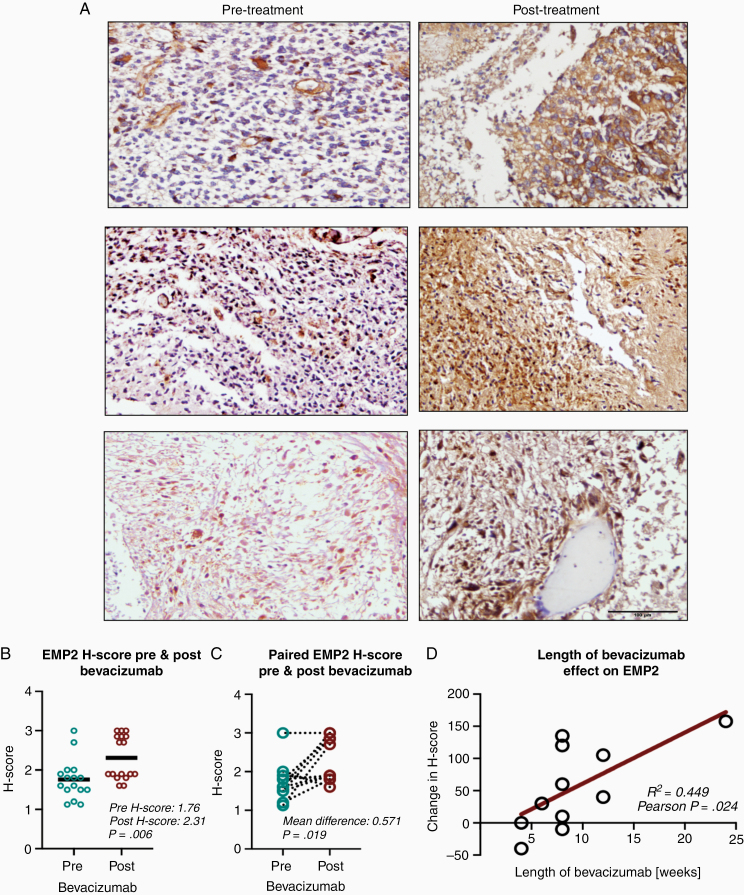
Bevacizumab therapy increases EMP2 levels. (A) Protein expression of EMP2 was visualized using standard IHC in clinical samples before and following bevacizumab treatment and eventual resistance. The expression pattern from 2 patients is shown. Magnification: 200×. Scale bar = 100 µm. (B and C) Protein expression of EMP2 in clinical samples increased after bevacizumab therapy in both unpaired (B) and paired (C) analyses. (D) Increase in protein expression was positively correlated with length of bevacizumab therapy.

EMP2 H-scores from 16 tumor samples obtained from patients undergoing initial resection for newly diagnosed glioblastoma were compared to EMP H-scores obtained from 17 patients undergoing subsequent resection after bevacizumab treatment for recurrent glioblastoma (Figure 2A). EMP2 H-scores were significantly higher in recurrent tumor samples after bevacizumab treatment (*mean H-score 2.31 vs 1.76; P = .006*; Figure 2B). A cohort of 12 patients had paired tumor tissue obtained from initial resection for newly diagnosed glioblastoma as well as from recurrent tumor resection after bevacizumab treatment. EMP2 H-scores were significantly higher in recurrent tumor samples after bevacizumab treatment relative to paired initial tumor samples (*mean difference 0.571; P = .019*; Figure 2C). We evaluated whether the length of bevacizumab treatment affected changes in EMP2 H-score before and after bevacizumab treatment. There was a positive linear correlation between length of bevacizumab treatment and change in EMP2 H-score (*R*^2^  *= 0.449; Pearson P = .024*; Figure 2D). Two patients were treated with medications that target angiogenesis in addition to bevacizumab prior to recurrent surgery: 1 patient with aflibercept (VEGF trap) and 1 patient with ofranergene obadenovec (VB-111). While not statistically powered, these patients interestingly showed increased H-scores relative to those without dual treatment (*mean H-score 300 vs 220; P = .063*).

## Discussion

Following accelerated approval from the US Food and Drug Administration in 2009, bevacizumab showed improved response rates and 6-month progression-free survival compared to historical controls, but since then, it has been shown that most patients’ tumors progress after a median time of 3–5 months.^4,28^ Given these results, novel methods are needed to quickly detect bevacizumab resistance as well as perhaps serve as a novel target for combination therapy. We have previously identified EMP2 as a protein associated with angiogenesis in glioblastoma^22^ and shown in glioblastoma cell lines that overexpression of this protein leads to increased tumor vascularity. In this study, we attempt to build upon our laboratory work with an investigation of EMP2 in clinical glioblastoma samples, and we specifically examine the effects of EMP2 following antiangiogenic treatment.

In this study, increased levels of EMP2 were observed in clinical tumor samples after bevacizumab treatment in both unpaired and paired analyses. We find that this increase in EMP2 levels correlated with the length of bevacizumab treatment and the presence of additional VEGF targeting therapeutics. We have previously shown that EMP2 may at least partially serve to increase tumor vascularity through upregulation of VEGF-A within the tumor parenchyma.^22^ Resistance to bevacizumab is a well-studied topic, with hypoxia-dependent and hypoxia-independent recruitment of alternative kinase signaling pathways,^29^ changes in autophagy,^30,31^ and upregulation of alternative promotors of angiogenesis^32–38^ as potential mechanisms for resistance. Interestingly, in previous gene expression studies in tumors before and after bevacizumab, EMP2 has not been identified as a highly upregulated gene.^38–41^ In this study, we focus on protein expression of this cell surface molecule and see consistent increases in expression after bevacizumab treatment.

Furthermore, differences in progression-free and overall survival in patients correlated with altered levels of EMP2 from tumor tissue after resection of newly diagnosed glioblastoma. Specifically, higher levels of EMP2 were associated with decreased survival following bevacizumab treatment. While the numbers tested are relatively small, we hypothesize that EMP2 levels may correlate with the efficacy of bevacizumab treatment. This will need to be confirmed by comparing survival to EMP2 levels in patients without bevacizumab treatment.

Lastly, we find an association between areas of vascular proliferation and EMP2 expression, suggesting increased utilization of EMP2 in glioma cells surrounding areas of angiogenesis. This has consequences for potential therapeutic delivery of anti-EMP2 agents as the target protein resides in areas around abnormal blood vessels without an intact blood–brain barrier. Overall, we attempt to build upon our previous work investigating EMP2 as a potential antiangiogenic therapy. We hypothesize that the EMP2 correlation to bevacizumab treatment may warrant future study as a potential mechanism for bevacizumab resistance and antiangiogenic therapy.

Finally, we note several limitations to the study. This study is susceptible to confounding bias given (1) small sample size and (2) heterogeneity in treatment including a few patients with therapy beyond the standard of care chemotherapy and radiation therapy (ie, immunotherapy). In addition, given all patients received temozolomide and radiation therapy, it is unclear whether these have an effect on EMP2 expression and is not specifically tested in our study. In addition, EMP2 may increase in recurrent tumors independent of treatment modalities. Lastly, given the intratumoral heterogeneity with EMP2 expression, EMP2 expression levels may be biased depending on the areas of sample selection. Despite these limitations, we believe this clinical data translate previous work in cell lines to highlight the potential of EMP2 as a biomarker for antiangiogenic therapy in glioblastoma.

## Conclusions

EMP2 is a cell surface protein found in glioblastoma that serves to increase angiogenesis in cell line models. High levels of EMP2 are associated with decreased survival after bevacizumab therapy. After bevacizumab therapy, EMP2 protein expression levels increase in a manner proportional to the length of therapy, and specifically, protein expression was enriched in areas of angiogenesis in glioblastoma. Overall, we hypothesize that these data suggest further study on EMP2 in the treatment response and resistance to bevacizumab as well as further evaluation as a tool to assess antiangiogenic therapies in glioblastoma.

## Notes

**Authorship statement:** Conception of study: K.P., L.G., T.C., L.L., W.Y., I.Y., and M.W; acquisition of data: K.P., S.K., S.T., C.D., A.M., L.L., W.Y., I.Y., and M.W; analysis of data: K.P., S.K., S.T., C.D., A.M., L.L., W.Y., I.Y., and M.W; drafting of manuscript: K.P., S.K., C.D., I.Y., and M.W; critical review of manuscript: all authors; study supervision: L.G., T.C., L.L., W.Y., I.Y., and M.W.
